# S07-4: How to promote HEPA in local urban communities in an eco-friendly way? Learn more about City, Green, Go! Toolkit

**DOI:** 10.1093/eurpub/ckae114.230

**Published:** 2024-09-26

**Authors:** Réka Veress

**Affiliations:** Budapest Sport Service Provider Nonprofit Ltd

## Abstract

**Purpose:**

The purpose of the City, Green, Go! project is to promote environmental sustainability in the grassroots sport sector. The focus is on promoting health-enhancing physical activities in the urban environment, that are taking into consideration the physical characteristics of the environment and spreading environmental messages. For empowering the grassroots sports’ actors, a toolkit has been developed according to the needs of municipalities, sport and environmental organisations, local communities to provide capacity building and a path for collaborative work.

**Project description:**

In the framework of a 2-year-long Erasmus+ sport collaborative partnership project, 5 main partners and an associated municipality have developed the City, Green, Go! Toolkit, the Toolkit for eco-friendly grassroots sports initiatives in urban settings.

With the coordination of Budapest Sport Service Provider Non-profit Ltd, Isinnova research institute has led the research and the collection of relevant good practices, which are available on the project website. Based on the good-practice compilation, the International Sport and Culture Association (ISCA) has led the development of the Toolkit, focusing on the following chapters:

• Why should we promote eco-friendly grassroots sport initiatives in urban settings?

• What can eco-friendly grassroots sport initiatives bring to society?

• Key steps to be taken to create eco-friendly grassroots sport initiatives in urban settings

• Useful Resources

Sport and Sustainability International (SandSI) coordinates the organisation of online and onsite testing workshops, that are for disseminate and further develop the toolkit. These workshops are used also as evaluation platforms. Based on the feedback of the participants, the toolkit will be improved.

The Toolkit is based on Principles for Environmental Conservation and Regeneration in Sport Events. Biodersity, Social Equity, Health and Wellness are also addressed among the principles.

For the effective use of the Toolkit, itineraries for organisations of online and onsite workshops will be created.

Dissemination is partly done during the workshops and in the framework of cooperation with like-minded Erasmus+ projects, which creates synergies among similar actions.

**Conclusions:**

By learning about the Principles for Environmental Conservation and Regeneration in Sport events, the environment and the members of the local communities will jointly benefit from eco-friendly grassroots sport events.

